# Gender Differences Influence Gender Equality Awareness, Self-Esteem, and Subjective Well-Being Among School-Age Children in China

**DOI:** 10.3389/fpsyg.2021.671785

**Published:** 2022-01-14

**Authors:** Yifei Li, Man Zuo, Yirong Peng, Jie Zhang, Yiping Chen, Yingxiang Tao, Biyun Ye, Jingping Zhang

**Affiliations:** ^1^Nursing Psychology Research Center, Xiangya School of Nursing, Central South University, Changsha, China; ^2^Heyuan People’s Hospital, Heyuan, China; ^3^Guijing Primary School, Changsha, China

**Keywords:** school-age children, gender, gender equality awareness, self-esteem, subjective well-being

## Abstract

The objective of this study was to investigate and analyze the status and influential factors of gender equality awareness, self-esteem, and subjective well-being in school-age boys and girls. The results can help schools and teachers provide more effective gender equality and mental health education. In the study, 284 valid questionnaires were collected from a total of 323 school-age boys and girls in the Hunan Province, China (effective response rate of 87.93%). The questionnaire covered gender equality awareness, self-esteem, and subjective well-being, with the influencing factors analyzed through multiple linear regression. There was a significant correlation among children’s gender equality awareness in all areas examined (family, occupation, and school), with both boys and girls having the lowest awareness of gender equality in occupational fields. The children’s self-esteem and subjective well-being were significantly correlated as well. Gender equality awareness, self-esteem, and subjective well-being among boys and girls reflected different influential factors. Androgynous traits (neither feminine nor masculine) were conducive to the development of gender equality awareness and self-esteem among the children. Therefore, schools and teachers need to provide gender equality and mental health education according to the specific psychological characteristics of each boy and girl.

## Introduction

Many studies have found that there are significant differences in gender equality awareness and mental health among different genders (Tennant et al., 2007; Tang et al., 2011). Men usually have lower gender equality awareness (Tang et al., 2011), whereas women usually have lower mental health (Tennant et al., 2007). Lower gender equality perceptions and lower mental health not only create psychological problems, such as anxiety and depression (Derdikman-Eiron et al., 2011; Holter, 2014), but also can endanger one’s physical health (Clarke et al., 2011; Holter, 2014) and increase the risk of death (Happell et al., 2017; Kolip et al., 2019). Studies have found that a high sense of gender equality can promote both men’s and women’s physical, mental, and sexual health (Bates et al., 2009; Syed, 2017); the higher the national gender equality index and personal mental health level, the longer the life expectancy of men and women (Kolip and Lange, 2018; Zaninotto and Steptoe, 2019; Gadoth and Heymann, 2020).

Common measures of individual mental health include self-esteem and subjective well-being (Derdikman-Eiron et al., 2011; Joshanloo and Daemi, 2015). As an important part of a healthy personality, self-esteem can affect both positive and negative psychological states of an individual (Nartova-Bochaver et al., 2019). Moreover, it is regarded as an important measure of mental health (Jin, 2014). Subjective well-being is a comprehensive evaluation of personal life according to subjective standards, which combine emotional experiences with life satisfaction (Wang, 2021), and is significantly correlated with 24 positive personality traits such as love and gratitude (Zhou and Liu, 2011). This is the most common positive psychological experience related to mental health (Derdikman-Eiron et al., 2011). High self-esteem and subjective well-being are associated with higher mental health (Derdikman-Eiron et al., 2011). An egalitarian perception of gender awareness leads to a higher sense of self-esteem and subjective well-being for both men and women (Schmitt et al., 2017). Men generally have higher self-esteem and subjective well-being than women, especially in areas with high gender equality (Derdikman-Eiron et al., 2011; Schmitt et al., 2017).

Most adult gender equality awareness and psychological problems stem from childhood, especially at school age (Rutter et al., 2006; Solbes-Canales et al., 2020). School age refers to the period of development from primary school to adolescence, usually between 6 and 12 years of age (Cui, 2013). At this stage, children are in a critical development period of individual gender equality awareness, self-esteem, and subjective well-being. Solbes-Canales et al. (2020) found gender stereotypes in children aged 4–9 years old and significant differences in different gender groups. Trautner et al. (2005) found that individual gender stereotypes appeared at the age of five, reached a peak of rigidity at 7 or 8 years of age, and then gradually gained flexibility as children’s gender cognition and understanding deepened. Zhang’s (2004) research on children aged 3–9 years old found that 4–5 years old and 7–8 years old were two key transitional periods for children’s self-esteem development. Casas and González-Carrasco’s (2019) analysis of children aged 7–14 in 15 countries found that in most countries, 10 years old was the transitional period for children’s subjective well-being. Moreover, significant differences in self-esteem and subjective well-being among school-age children of different genders have been found (Zhang, 2004, 2012; Savoye et al., 2015). Studies point out that higher gender equality, self-esteem, and subjective well-being can improve the mental health and life satisfaction of school-age children (Zhang, 2012; Looze et al., 2018). Overall, school age is a critical development period for gender equality awareness, self-esteem, and the subjective well-being among children. Positive gender equality awareness, self-esteem, and subjective well-being can have a positive impact on school-age children, and there are significant differences in gender equality awareness, self-esteem, and subjective well-being among children of different genders.

In general, biological and sociological theories are used to explain the psychological and behavioral differences among children of different genders (Chaplin and Aldao, 2013; Chen, 2016). According to biological theories, gender differences in psychology and behavior are caused by congenital factors, such as sex hormones, chromosomes, genes, and heredity (Feingold, 1994; Chaplin and Aldao, 2013). According to sociological theories, children’s gender equality awareness and role development are formed through social learning and cognition (Chaplin and Aldao, 2013; Chen, 2016). Children of different genders will have different gender role responses in different or similar groups (Rose and Rudolph, 2006; Chen, 2016). Gender roles refer to the gender characteristics that a society assigns to men and women (Bem, 1981). For example, men tend to be seen as independent, aggressive, and ambitious, whereas women are seen as affectionate, gentle, and compassionate (Bem, 1974). “Masculinity” refers to individuals who have highly masculine characteristics and “femininity” refers to individuals who have highly feminine characteristics; individuals with both highly masculine and highly feminine traits are considered “androgynous.” Those who have both low masculine and low feminine characteristics are considered “undifferentiated” (Bem, 1974). Studies have shown that androgynous profiles predict positive outcomes, such as greater self-esteem and mental health (Arcand et al., 2020).

However, with further investigation in many studies, more and more scholars have found that gender differences in individual psychological and behavioral responses are influenced by a wide variety of factors (Brody, 1999; Fang, 2010). Consequently, Wood and Eagly (2002) proposed a new concept called the biosocial model. This model posits that the differences in gender equality awareness and psychological behavior responses between men and women are caused by social, cultural, and environmental interventions along with biological factors such as genes and hormones, mainly influenced by congenital factors, critical development periods, and parenting (Chen, 2016). Therefore, under the premise that biological factors cannot be changed, understanding the key development periods for children’s gender equality awareness and psychology, targeted, reasonable education, and guidance for children of different genders have become a research focus.

According to several scholars, the transitional periods of children’s gender equality awareness, self-esteem, and subjective well-being appear at school age (Zhang, 2004; Trautner et al., 2005; Casas and González-Carrasco, 2019). Moreover, some have found that school and teacher support are more conducive than family and other social support in enhancing children’s gender equality, self-esteem, and subjective well-being (Chu et al., 2010; Weber et al., 2010; Chen, 2016). Hence, a positive educational environment can also effectively promote children’s physical and mental health, along with their academic development (Atkins et al., 2017). Therefore, during primary education, schools and teachers should help children establish gender equality awareness, self-esteem, subjective well-being, and other positive psychological states through education and interventions, which will be critical for their future physical and mental health and also their academic success.

Compared with the United States, Sweden, Taiwan, and other regions (Li, 2007; Liu, 2015; Brener and Demissie, 2018; Okenwa-Emgwa and von Strauss, 2018), gender equality and mental health education on the Chinese mainland started late and is still in an exploratory stage. Relevant courses and activities have been conducted only in some regions and schools, with short follow-up. Moreover, there are few studies on gender equality awareness, self-esteem, and subjective well-being among Chinese school-age boys and girls. This study aims to fill this gap by providing theoretical and educational guidance for education and intervention in schools through the investigation and analysis of gender differences in school-age children’s gender equality awareness, self-esteem, and subjective well-being in China. Our objective is to help teachers and other practitioners to improve gender equality awareness, self-esteem, and subjective well-being among school-age children.

## Materials and Methods

### Design and Sample

Participants were recruited from two primary schools in Hunan Province, China, through convenience sampling from June 15, 2020 to June 19, 2020. The screening criteria were as follows: (1) students had to be in grades first through sixth and were willing to voluntarily participate in the study; (2) had their parents’ consent; and (3) had not participated in other studies on gender equality and mental health of children.

The principal of the research group contacted the cooperative schools in advance of the study. After unified training, a researcher and a class teacher introduced the study’s purpose, content, and questionnaire terms to the children. After obtaining consent from the children and parents, the questionnaire was distributed and collected on-site. The children were free to ask questions, and members of the research team explained questions that the children did not understand.

Power analysis using the GPower software showed that a minimum of 210 participants were required to achieve a significant outcome (Effect size *d*: 0.5, α error prob.: 0.05, Power: 0.95). A total of 323 questionnaires were returned and 284 were valid in this study, with a recovery rate of 87.93%.

### Measurements

The questionnaire consisted of the following five sections.

#### Demographic Characteristics

The demographic data section collected the following: gender, age, grade, number of same-sex friends, number of opposite-sex friends, whether the child had siblings, whether the child had been left-behind, parental marital status, frequency of parental quarrels, children’s gender satisfaction, and attitudes toward gender-neutral dressing for boys and girls.

#### Gender Equality Awareness Questionnaire

The gender equality awareness section was developed by Tang et al. (2011). The questions involved attitudes toward gender equality in family fields (e.g., who do you think should do the cooking?), school fields (e.g., who do you think the teacher should give more chances to act as a monitor?), and occupational fields (e.g., who do you think is more suitable to be a kindergarten teacher?). Each area contained 10 items, for a total of 30 items. For scoring, option B (same for men and women) was coded as 1, and options A (more suitable for men) and C (more suitable for women) were both coded as 0. The higher the total score, the stronger the consciousness of gender equality. Cronbach’s alpha was 0.92 here.

#### Bem Sex Role Inventory

The Bem Sex Role Inventory (BSRI) was designed by Bem (1974) and translated by Lu and Su (2003) to measure gender role orientation. It was scored on a 7-point Likert-type scale where 1 represented “never true” and 7 “always true.” It contained three subscales: a 14-item masculinity scale (e.g., ambitious and aggressive), a 12-item femininity scale (e.g., gentle and affectionate), and a 20-item gender-neutral scale (e.g., helpful and happy). The total score of the masculinity scale was divided by 14, and the total score of the femininity scale was divided by 12. If the scores of both masculine and feminine items were greater than four, the measurement result was considered androgynous. If only the masculinity scale was higher than four, the result was masculine; if only the femininity scale score was higher than four, the result was feminine; if both masculinity and femininity scales’ scores were less than four, the result was undifferentiated. The BSRI had an internal consistency and test–retest reliability of around 0.80 (Lu and Su, 2003). Cronbach’s alpha was 0.92 here.

#### Rosenberg Self-Esteem Scale

The Rosenberg Self-esteem Scale (RSES) was designed by Rosenberg (Lee, 1969) and translated by Yu and Ji (1993). It is the most widely used questionnaire to assess self-esteem. The scale includes 10 items, scored on a 4-point Likert-type scale ranging from 1 (strongly disagree) to 4 (strongly agree). The overall self-esteem factor was calculated by a sum score ranging from 10 to 40; higher scores indicated higher self-esteem. Cronbach’s alpha was 0.78 for the Chinese version (Yu and Ji, 1993) and 0.62 here.

#### Subjective Well-Being

The subjective well-being measurement, designed by Andrews and Withey (Lorish and Maisiak, 1986) and translated by Zhang (2010), was a simplified face scale. This brief and pictorial mood scale used a sequence of seven faces that did not require reading literacy. Cronbach’s alpha was 0.7 for the overall scale.

### Ethical Considerations

The study was reviewed by the Institutional Review Board in the researchers’ university (ethical grant number: E201947). Prior to the survey, the researchers informed subjects of the purpose, method, and considerations of the study, and they could quit at any time during the filling process. Researchers issued the anonymous questionnaire to all eligible children after acquiring written informed consent. A quiet and private meeting room was provided for participants to complete the questionnaire. Questionnaires were retrieved immediately, and only the researchers had access to the data. The cover page of the questionnaire contained contact information of psychological consultation.

### Data Analysis

SPSS software (version 21.0) was used for statistical analysis. Categorical data were expressed as frequencies and percentages. Measurement data were expressed as mean ± SD. An independent sample *t*-test and one-way ANOVA were used to analyze the demographic data of gender equality awareness, self-esteem, and subjective well-being. The significant variables in the univariate analysis were included in the multiple linear regression equation. Gender equality awareness, self-esteem, and subjective well-being of school-age children were analyzed through multiple factors. Incorporating all possible variables, we used multiple stepwise regression to analyze the factors influencing gender equality awareness, self-esteem, and the subjective well-being of children of different genders. Statistical significance was set at *p* < 0.05.

## Results

### Participant Demographics

Among the 284 participants, 133 were boys and 151 were girls, with a mean age of 9.49 (SD = 1.716, range = 6–13) years old. Most participants had siblings (76.06%), had not been left-behind (66.90%), had very good parental feelings (55.99%), had parents who did not quarrel (46.48%) or quarreled little (46.83%), had more than three same-sex (62.68%) and opposite-sex friends (36.62%), were satisfied with their gender (49.65%), and were categorized as androgynous (59.51%). As for the attitudes of the children toward gender-neutral dress for boys and girls, 44.02% did not like gender-neutral dressing among boys, 44.01% did not like gender-neutral dressing among girls, and 44.72% did not care about gender-neutral dressing among girls (see Table 1).

**TABLE 1:** Relationships among socio-demographic characteristics and variable scores (*N* = 284).

Variables	*N* (%)	Gender equality awareness	*t*/*F*	*p*	Self-esteem	*t*/*F*	*p*	Subjective well-being	*t*/*F*	*p*
**Gender**										
Male	133 (46.83)	15.86 ± 8.31*	−2.843	0.005	27.95 ± 5.01	0.720	0.472	2.19 ± 1.31	−1.311	0.191
Female	151 (53.17)	18.55 ± 7.60			27.55 ± 4.47			2.41 ± 1.56		
**Grade**										
1	17 (5.98)	8.82 ± 7.40**	16.828	0.000	25.41 ± 4.71*	2.304	0.045	2.18 ± 0.69	0.338	0.890
2	69 (24.30)	14.78 ± 7.14			26.99 ± 4.00			2.15 ± 1.53		
3	55 (19.37)	14.55 ± 5.71			28.58 ± 3.52			2.28 ± 1.51		
4	37 (13.03)	17.86 ± 8.36			27.16 ± 5.28			2.41 ± 1.55		
5	48 (16.90)	19.17 ± 7.37			27.81 ± 5.15			2.36 ± 1.29		
6	58 (20.42)	23.00 ± 7.10			28.83 ± 5.47			2.45 ± 1.56		
**The only child**										
Yes	68 (23.94)	17.18 ± 8.52	−0.136	0.892	28.16 ± 5.18	0.844	0.388	2.36 ± 1.47	0.338	0.736
No	216 (76.06)	17.33 ± 7.90			27.61 ± 4.58			2.29 ± 1.45		
**The left-behind children**										
Yes	94 (33.10)	16.57 ± 7.77	−1.058	0.291	27.13 ± 4.57	−1.538	0.125	2.29 ± 1.36	−0.160	0.873
No	190 (66.90)	17.65 ± 8.17			28.04 ± 4.78			2.317 ± 1.498		
**Parents’ feeling**										
Good feelings	159 (55.99)	17.94 ± 8.14	2.13	0.97	28.40 ± 4.64*	2.98	0.32	2.03 ± 1.29*	5.447	0.001
Average	87 (30.63)	16.84 ± 7.29			27.14 ± 4.44			2.79 ± 1.57		
Bad feelings	14 (4.93)	13.14 ± 7.29			25.43 ± 5.39			2.26 ± 1.31		
Divorce	24 (8.45)	15.43 ± 8.31			26.74 ± 5.03			2.62 ± 1.67		
**Parental quarrel**										
Never	132 (46.48)	17.95 ± 8.02	1.764	0.173	28.17 ± 7.73	2.089	0.126	2.04 ± 1.29**	11.459	0.000
Few	133 (46.83)	16.19 ± 7.63			27.56 ± 4.54			2.40 ± 1.42		
Many	19 (6.69)	18.21 ± 8.75			25.89 ± 5.374			3.66 ± 1.89		
**Number of same-sex friends**										
0	18 (6.34)	14.53 ± 8.87	1.138	0.334	27.29 ± 3.84*	4.836	0.003	2.095 ± 1.30*	3.276	0.022
1	35 (12.32)	16.35 ± 7.89			25.38 ± 5.36			3.04 ± 1.67		
2−3	53 (18.66)	16.31 ± 7.28			26.94 ± 3.40			2.26 ± 1.41		
>3	178 (62.68)	17.65 ± 8.03			28.44 ± 4.82			2.22 ± 1.40		
**Number of opposite-sex friends**										
0	77 (27.11)	16.82 ± 8.31	0.802	0.494	27.72 ± 4.99	2.358	0.072	2.52 ± 1.50	1.818	0.144
1	54 (19.02)	16.50 ± 8.76			26.65 ± 3.50			2.561 ± 1.54		
2−3	49 (17.25)	16.04 ± 7.55			27.02 ± 5.93			2.22 ± 1.53		
>3	104 (36.62)	17.97 ± 7.40			28.57 ± 4.27			2.32 ± 1.45		
**Gender satisfaction**										
Dissatisfied	36 (12.68)	14.50 ± 6.79**	10.421	0.000	26.33 ± 4.81	1.873	0.156	1.99 ± 1.44	2.195	0.113
Indifferent	107 (37.67)	19.93 ± 8.28			28.04 ± 27.74			2.19 ± 1.48		
Satisfied	141 (49.65)	16.00 ± 7.64			27.87 ± 4.19			2.48 ± 1.42		
**Boy gender-neutral**										
Dislike	125 (44.02)	15.01 ± 7.65**	10.701	0.000	27.90 ± 4.29	0.991	0.372	2.26 ± 1.49	1.425	0.242
Indifference	82 (28.87)	19.96 ± 7.84			28.09 ± 4.66			2.52 ± 1.36		
Like	77 (27.11)	18.16 ± 7.92			27.10 ± 5.43			2.16 ± 1.48		
**Girl gender-neutral**										
Dislike	125 (44.01)	16.40 ± 7.72	1.179	0.309	27.81 ± 4.57	0.483	0.618	2.39 ± 1.48	1.768	0.173
Indifference	127 (44.72)	18.01 ± 8.28			27.74 ± 4.53			2.29 ± 1.42		
Like	32 (11.27)	17.17 ± 7.71			28.63 ± 4.61			1.84 ± 1.14		
**Sex role**										
Undifferentiated	45 (15.85)	14.58 ± 7.68**	6.836	0.000	26.02 ± 4.65*	3.270	0.022	2.21 ± 1.42	2.367	0.071
Masculinity	26 (9.15)	14.54 ± 7.65			27.77 ± 4.46			2.58 ± 1.70		
Femininity	44 (15.49)	15.02 ± 7.33			27.11 ± 5.45			2.77 ± 1.30		
Androgynous	169 (59.51)	19.03 ± 7.97			28.36 ± 4.49			2.17 ± 1.44		

**p = 0.00, *p < 0.05, these scores were significantly higher or lower than those for other groups within the socio-demographic characteristics.

### Gender Equality Awareness, Self-Esteem, and Subjective Well-Being of School-Age Children

Table 2 shows the descriptive analysis of gender equality awareness, self-esteem, and subjective well-being of school-age children. The children’s average score for gender equality awareness was 17.29 ± 8.04, gender equality awareness of family fields was 6.32 ± 2.73, gender equality awareness of occupational fields was 5.14 ± 3.20, gender equality awareness of school fields was 5.84 ± 3.21, self-esteem was 27.74 ± 4.73; and subjective well-being was 2.31 ± 1.45. We correlated the scores of gender equality awareness for family, school, and occupational fields, and the self-esteem and subjective well-being scores (see Table 3).

**TABLE 2:** Descriptive statistics of the measured variables (*N* = 284).

Variables	*Mean*	SD	Median	Range
Gender equality awareness	17.29	8.04	17	0−30
Family fields	6.32	2.73	6	0−10
Occupational fields	5.14	3.20	5	0−10
School fields	5.84	3.21	6	0−10
Self-esteem	27.74	4.73	27	10−40
Subjective well-being	2.31	1.45	2	1−7

**TABLE 3:** Correlations among the measured variables (*N* = 284).

	Gender equality awareness	Family fields	Occupational fields	School fields	Self-esteem	Subjective well-being
Gender equality awareness	1					
Family fields	0.851**	1				
Occupational fields	0.905**	0.683**	1			
School fields	0.878**	0.601**	0.689**	1		
Self-esteem	0.083	0.102	0.026	0.094	1	
Subjective well-being	0.036	0.017	0.019	0.058	−0.185**	1

**p = 0.00.

**FIGURE 1:**
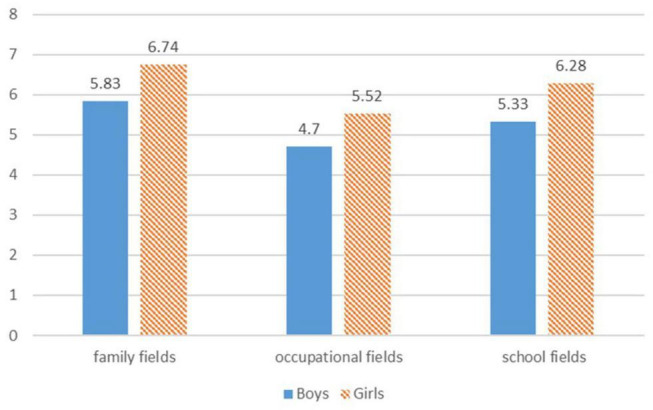
Gender differences of gender equality awareness in all fields with school-age children.

**FIGURE 2:**
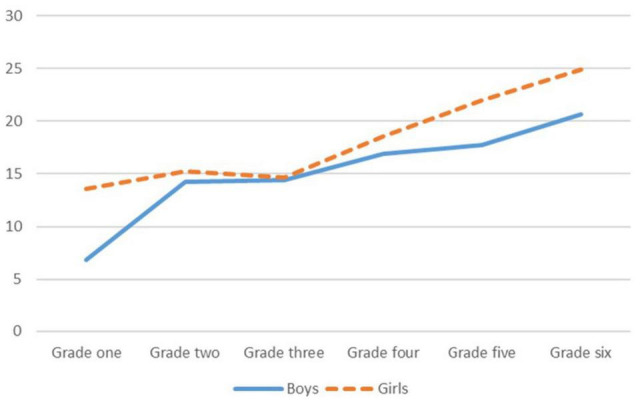
Variation trend of overall gender equality awareness in different grades.

Figure 1 shows the average score of gender equality awareness among children of different genders in all fields. Both boys and girls scored lower in occupational fields than in family and school fields. Figure 2 shows that the overall gender equality awareness of boys and girls increased with each grade.

### Analysis on the Influential Factors of School-Age Children’s Gender Equality Awareness

Table 4 shows that gender, grade, gender role, and attitude toward gender-neutral dressing for boys were the main factors influencing gender equality awareness among all children. Table 5 shows that the main factors influencing gender equality awareness among the boys were grade, whether they were an only child, gender satisfaction, and their attitude toward gender-neutral dressing for boys. The main factors influencing gender equality awareness among the girls were grade, whether they were an only child and whether they were left-behind, the number of opposite-sex friends, and gender roles.

**TABLE 4:** Multiple linear regression analysis of the study variables on gender equality awareness.

Dependent variable	Independent variables	*B*	*Beta*	*t*	*p*
Gender equality awareness	(Constant)	1.580		0.882	0.378
	Gender	2.204	0.137	2.692	0.008
	Grade	2.010	0.405	7.711	0.000
	Gender role	1.411	0.148	2.826	0.005
	Attitudes toward boys’ gender-neutral dressing	1.032	0.146	2.826	0.005

*R = 0.284, F = 27.628, p = 0.000.Significant variables in univariate analysis were included, and multiple linear regression analysis was used to predict the related influential factors of gender equality awareness. Assumed predictors: gender, grade, gender satisfaction, attitudes toward boys’ gender-neutral dressing, gender role.*

**TABLE 5:** Multiple linear stepwise regression analysis of the study variables on gender equality awareness among boys and girls.

Dependent variable	Independent variables	BOYS^1^				GIRLS^2^			
		*B*	*Beta*	*t*	*p*	*B*	*Beta*	*t*	*p*
Gender equality awareness	(Constant)	–4.218		–1.106	0.271	5.401		1.352	0.179
	Attitudes toward boys’ gender-neutral dressing	2.123	0.212	2.591	0.011				
	Gender satisfaction	2.254	0.185	2.238	0.027				
	grade	1.684	0.335	4.001	0.000	2.428	0.493	6.624	0.000
	The only child	3.014	0.167	2.071	0.041	–2.892	–0.146	–1.998	0.048
	The left-behind children					2.269	0.143	1.981	0.050
	Number of opposite-sex friends					0.988	0.168	2.302	0.023
	Gender role					1.223	0.179	2.431	0.016

*^1^R^1^ = 0.262, F = 10.118, p = 0.000.*

*^2^R^2^ = 0.352, F = 13.923, p = 0.000.*

*All possible variables were included, and stepwise multiple linear regression analysis was used to predict the related influential factors of gender equality awareness among children with different genders. Assumed predictors: gender, grade, the only child, the left-behind children, parents’ feeling, parents quarrel, number of same-sex friends, number of opposite-sex friends, gender satisfaction, attitudes toward boys’ gender-neutral dressing, attitudes toward girls’ gender-neutral dressing, gender role.*

### Analysis on the Influential Factors of School-Age Children’s Self-Esteem

Table 6 shows that gender role, parental feelings, and number of same-sex friends were the main factors influencing self-esteem. Table 7 shows that parental feelings were the main factor that affect the boys’ self-esteem, whereas gender role was the main factor that affect the girls’ self-esteem.

**TABLE 6:** Multiple linear regression analysis of the study variables on self-esteem.

Dependent variable	Independent variables	*B*	*Beta*	*t*	*p*
Self-esteem	(Constant)	25.020		19.284	0.000
	Gender role	0.550	0.133	2.236	0.026
	Parents’ feeling	–0.659	–0.128	–2.150	0.032
	Number of same-sex friends	0.776	0.153	2.551	0.011

*R = 0.071, F = 6.759, p = 0.000.*

*Significant variables in univariate analysis were included, and multiple linear regression analysis was used to predict the related influential factors of self-esteem. Assumed predictors: grade, parents’ feeling, number of same-sex friends, gender role.*

**TABLE 7:** Multiple linear stepwise regression analysis of the study variables on self-esteem among boys and girls.

Dependent variable	Independent variables	BOYS^1^				GIRLS^2^			
		*B*	*Beta*	*t*	*p*	*B*	*Beta*	*t*	*p*
Self-esteem	(Constant)	30.085		32.611	0.000	25.596		31.191	0.000
	Parents’ feeling	–1.214	–0.233	–2.588	0.011				
	Gender role					0.967	0.328	2.946	0.004

*^1^R^1^ = 0.054, F = 6.695, p = 0.011.*

*^2^R^2^ = 0.062, F = 8.682, p = 0.004.*

*All possible variables were included, and stepwise multiple linear regression analysis was used to predict the related influential factors of self-esteem among children with different genders. Assumed predictors: gender, grade, the only child, the left-behind children, parents’ feeling, parents quarrel, number of same-sex friends, number of opposite-sex friends, gender satisfaction, attitudes toward boys’ gender-neutral dressing, attitudes toward girls’ gender-neutral dressing, gender role.*

### Analysis on the Influential Factors of School-Age Children’s Subjective Well-Being

In Tables 8, 9, we show that subjective well-being was affected mainly by the extent of parental quarrels, which was more harmful for girls.

**TABLE 8:** Multiple linear regression analysis of the study variables on subjective well-being.

Dependent variable	Independent variables	*B*	*Beta*	*t*	*p*
Subjective well-being	(Constant)	1.374		5.757	0.000
	Parental quarrel	0.590	0.251	4.255	0.000

*R = 0.063, F = 18.107, p = 0.000.*

*Significant variables in univariate analysis were included, and multiple linear regression analysis was used to predict the related influential factors of subjective well-being. Assumed predictors: parents’ feeling, parental quarrel, number of same-sex friends.*

**TABLE 9:** Multiple linear stepwise regression analysis of the study variables on subjective well-being among boys and girls.

Dependent variable	Independent variables	BOYS				GIRLS^2^			
		*B*	*Beta*	*t*	*p*	*B*	*Beta*	*t*	*p*
Subjective well-being	(Constant)					1.258		3.699	0.000
	Parental quarrel					0.750	0.306	3.698	0.000

*R^2^ = 0.094, F = 13.677, p = 0.000.*

*All possible variables were included, and stepwise multiple linear regression analysis was used to predict the related influential factors of subjective well-being among children with different genders. Assumed predictors: gender, grade, the only child, the left-behind children, parents’ feeling, parents quarrel, number of same-sex friends, number of opposite-sex friends, gender satisfaction, attitudes toward boys’ gender-neutral dressing, attitudes toward girls’ gender-neutral dressing, gender role.*

## Discussion

In our study, the average scores for the children were as follows: gender equality awareness was 17.29 ± 8.04; self-esteem was 27.74 ± 4.73; and subjective well-being was 2.31 ± 1.45. Gender equality awareness across family, occupation, and school fields was significantly correlated. There was a significant correlation between self-esteem and subjective well-being as well. However, there was no correlation between gender equality awareness and self-esteem and subjective well-being. This may be due to the fact that our sample was small compared with previous studies that utilized large samples across borders or across regions (Campbell et al., 2021). We conducted a survey only in Hunan Province, China, and all the children were from the same gender, sociocultural environment.

### Analysis on the Status and Influential Factors of School-Age Children’s Gender Equality Awareness

In our study, as stated above, school-age children’s family, occupation, and school gender equality awareness scores were significantly correlated. Girls’ gender equality awareness was slightly higher than that of the boys’ overall gender equality awareness and in the various fields. However, both male and female students had the lowest gender equality awareness of the occupational field, whereas gender equality awareness was relatively high for family and school fields. This is consistent with the results of Tang et al. (2011). Moreover, this may relate to sex segregation in China’s occupational environment. Previous studies have found (e.g., Li, 2016) that compared with developed countries and other developing countries, China’s sex segregation is more significant. In China, 87.9% of professional women are engaged in low-grade, non-technical occupations, such as agriculture, forestry, service, and production, with the proportion of women in high-level occupations relatively low (Li, 2016). Both men and women in China are challenged by occupational gender stereotypes (Sun, 2010). This detrimental environment may have a negative impact on children’s occupational gender equality awareness and also their future employment choices. Therefore, schools and teachers should strive to cultivate gender equality awareness in children and eliminate gender stereotypes in classroom education and extracurricular activities. Specifically in terms of occupational fields, teachers should emphasize that occupational choices should be made based on personal interest and practical ability, but not on gender.

We found that gender, grade, gender role, and attitude toward gender-neutral dressing for boys were the main factors that influence gender equality awareness. Among that for girls, the oldest, those having androgynous traits, and those indifferent to gender-neutral dressing for boys had higher awareness of gender equality, consistent with previous results (Tang et al., 2011; Li, 2019). Except for the third grade, gender equality awareness among both boys and girls improved with each grade. This may relate to the fact that children in the third grade are generally 7–8 years old. Previous studies (e.g., Trautner et al., 2005) have shown that in the development of children’s gender equality awareness, children aged seven to eight are at the peak of gender stereotyping due to factors such as enhanced self-awareness and inadequate understanding of gender cognition. Subsequently, as children continue to learn and grow, their knowledge of gender expands and they are likely to exhibit more gender equality awareness (Trautner et al., 2005). Therefore, third-grade teachers need to pay special attention to cultivating the concept of gender equality, changing children’s gender stereotypes through gender equality themed classes and storytelling. At the same time, teachers of all grades should encourage children’s mixed-gender communication through sports and team assignments to cultivate children’s androgynous traits and educate children to respect their peers. In so doing, they can avoid prejudicing gender-neutral behavior and the appearance of boys and girls.

In our analysis, we found that girls falling under the androgynous category had higher gender equality awareness, whereas those in lower grades, who had siblings, had been left-behind, and had fewer opposite-sex friends, had lower gender equality awareness. For gender equality awareness among boys, those in a lower grade, who were only children, and unsatisfied with boys’ gender-neutral dressing had lower awareness. The different factors that affect gender equality awareness between male and female pupils, especially the “only child” difference, may relate to biological differences and the male-dominated social environment (Jiang et al., 2016; Polderman et al., 2018).

Most Chinese families have a preference for sons (Liao and Zhang, 2020). According to the Global Gender Gap Report 2018, China’s sex ratio at birth ranks second to last among 149 countries (Wang et al., 2020). A survey of pregnant women in China by Loo et al. (2009) found that most pregnant women are more likely to believe they are pregnant with a boy. In Liao and Lian’s (2020) study of mental health during China’s only child and then more than one child policy between 1995 and 2017, they found that boys and only children generally had better mental health and positive development due to greater family care. As a result, boys, especially those with siblings, received more preference and family support than girls did. Thus, as the preferred child in the family, male children with siblings not only had a higher level of mental health than female children with siblings, but also were more intuitive about the existence of gender inequality than boys who were only children. The positive psychology of bearing witness to one’s sister suffering from unequal treatment may produce a positive sense of gender equality among these boys. Female children with siblings may be influenced by an unequal family atmosphere since childhood, propagating gender inequality. Moreover, they may be too accustomed to some inequalities to fight back. The low gender equality awareness of only children who are boys may relate to an unintuitive perception of the harm brought to others by gender inequality. Females who are only children and who have received all the support from their family may pursue gender equality due to the psychological gap that they face in gender inequality outside the family. Therefore, schools and teachers should focus on observing gender inequality awareness among school-age boys who are only children and girls who have siblings to help them establish gender equality concepts. Teachers should communicate with families with multiple children through family visits, explaining the importance of gender equality to the parents and other family members, guiding them to treat their sons and daughters equally.

In addition, schools and teachers should encourage school-age boys to respect male gender-neutral behaviors and appearance and improve their gender satisfaction. For school-age girls, especially girls who were left-behind, they may have negative gender equality awareness due to the absence of parents during their growth (Wang, 2015). Therefore, teachers should give girls who were left-behind more care and teaching, increasing the contact between boys and girls by developing team games and other methods, cultivating children’s androgynous roles, helping them make friends with the opposite sex, respecting different genders, and striving to cultivate a more positive awareness of gender equality among the children.

### Analysis on the Status and Influential Factors of School-Age Children’s Self-Esteem and Subjective Well-Being

In our study, children scored well in terms of self-esteem and subjective well-being; these scores were also correlated; the higher their self-esteem, the higher their subjective well-being, which is consistent with the results of previous studies (Wang, 2013). Those categorized as androgynous children had higher self-esteem, which is also consistent with previous studies (Juster et al., 2016). This may be because these children possess both male and female gender traits, have better psychological adaptability and adjustment ability, and are, therefore, more likely to develop positive self-esteem (Lo et al., 2019). School-age children with more same-sex friends also had higher self-esteem, in line with the results of Wang (2013). The implication is that good peer relationships can promote self-esteem effectively. Therefore, while conducting gender education, schools and teachers should also help children become familiar with and communicate with more friends through group games and team tasks. These activities can help to promote children’s self-esteem and mental health. In addition, we found that children with poor parental feelings had lower self-esteem, along with children whose parents often quarreled. This reflects the importance of parents and the family environment on children’s mental health. Therefore, school teachers should also take advantage of opportunities, such as family visits and class meetings, to explain the psychological characteristics and needs of school-age children to parents and other guardians, guiding them in building a positive family atmosphere that can improve children’s self-esteem and subjective well-being.

Our analysis of the factors influencing self-esteem and subjective well-being among school-age children of different genders found that the self-esteem of girls is related mainly to gender roles and beneficial androgynous traits. This may relate to the positive effect of masculinity on children’s self-esteem (Lo et al., 2019). School-age boys’ self-esteem was affected mainly by their parental feelings. Boys with good parental feelings had higher self-esteem. Parental quarrels were more harmful to the subjective well-being of school-age girls; the more parents quarreled, the lower the subjective well-being of the girls. Therefore, when offering psychological guidance for children and communication with parents, schools and teachers should provide targeted education and guidance according to the influence of different gender roles and family conditions on the mental health of the boys and girls.

## Limitations

First, we investigated only the effects of some general demographic data and gender roles on gender equality awareness, self-esteem, and subjective well-being among school-age children. We did not, therefore, study children’s social support, internal psychological qualities, and more comprehensive demographic data. Second, we only surveyed children in two primary schools in the Hunan Province in China, which may cause sampling error. Third, our study has not sorted out a guiding theoretical framework. Future studies should expand the sample size to establish a more appropriate theoretical framework and test comprehensively the effects of internal and external factors on gender equality awareness, self-esteem, and the subjective well-being of school-age children.

## Conclusion

We investigated and analyzed the environmental situations and factors influencing gender equality awareness, self-esteem, and subjective well-being among school-age children of different genders. We found that gender equality awareness among school-age children in all areas was significantly correlated. Both boys and girls had the lowest scores for gender equality in occupational fields. Additionally, the factors that influence gender equality awareness, self-esteem, and subjective well-being among boys and girls differed. However, androgynous traits contributed to gender equality awareness and also self-esteem development in both boys and girls. In sum, schools and teachers should strengthen gender equality awareness of occupational fields and provide gender education and psychological counseling for each child. Specifically, schools and teachers should conduct intersex education to help children to develop strong androgynous temperaments to promote children’s gender equality awareness and self-esteem development.

## Data Availability Statement

The raw data supporting the conclusions of this article will be made available by the authors, without undue reservation.

## Ethics Statement

The studies involving human participants were reviewed and approved by the Xiangya School of Nursing, Central South University (Ethical Grant Number: E201947). Written informed consent to participate in the study was provided by the participants’ legal guardian/next of kin.

## Author Contributions

YL and MZ designed the study, collected the data, analyzed the data, interpreted the results, and wrote the manuscript. JZ conducted the statistical analysis and provided consultation in the study design and intervention development process. YC and YT interpreted the data, prepared the manuscript, and revised the manuscript. BY and YP conducted the statistical analysis and interpreted the result. JPZ designed the study, analyzed the data, interpreted the results, and provided consultation in the study design and intervention development process. All authors have read and reviewed the final manuscript.

## Conflict of Interest

The authors declare that the research was conducted in the absence of any commercial or financial relationships that could be construed as a potential conflict of interest.

## Publisher’s Note

All claims expressed in this article are solely those of the authors and do not necessarily represent those of their affiliated organizations, or those of the publisher, the editors and the reviewers. Any product that may be evaluated in this article, or claim that may be made by its manufacturer, is not guaranteed or endorsed by the publisher.
